# A Clinical Lecture on Psoriasis

**Published:** 1888-03

**Authors:** Henry J. Reynolds

**Affiliations:** Professor of Dermatology in the College of Physicians and Surgeons, Chicago, Illinois; Professor of Skin and Genito-Urinary Diseases, Chicago Polyclinic; Chief Dermatologist to the West Side Dispensary; Surgeon to the Department for Genito-Urinary Diseases, West Side Dispensary, Chicago, Illinois


### A CLINICAL LECTURE ON PSORIASIS.

---

BY HENRY J. REYNOLDS, M. D.,

Professor of Dermatology in the College of Physicians and Surgeons, Chicago, Illinois; Professor of Skin and Genito-Urinary Diseases, Chicago Polyclinic; Chief Dermatologist to the West Side Dispensary; Surgeon to the Department for Genito-Urinary Diseases, West Side Dispensary, Chicago, Illinois.

---

GENTLEMEN—These two little girls come to us to-day to be interviewed. I want to call your attention more particularly to the younger one, and may state before proceeding further that the case is one of "psoriasis." As many or most of you are not familiar with this disease, with its characteristics, general appearance, etc., I think it will be well, before examining this particular case, or studying the disease as it is manifested here, to first refresh your memory upon some of its general characteristics.

In the order of frequency, I think psoriasis ranks third or fourth, acne and eczema, of course, being more frequent than psoriasis, and possibly the syphilitic eruptions are more common than this disease. But it is sufficient to say, however, in regard to its frequency, that it is a very common affection. It is, of course, not contagious; it occurs about equally in both sexes, and may develop at any age, although it is more frequently encountered in early life—that is, in childhood or young adult life—very rarely in infancy, and likewise is rarely met with in old age. If we meet with it in old age we almost invariably have a history of its previous existence. The first manifestations of the disease are in the form of very small maculo-papular lesions, perhaps not larger than the size of a pin-head, covered by a thin scale or film. Now, while this is one of the characteristics of the disease, and the first one that manifests itself, it may be one that you do not often see, inasmuch as the disease has frequently passed through that stage of development before the case comes under your observation. When we find very many lesions of this character, it is sometimes described as *psoriasis punctata*, from the punctiform character of the lesions. As the disease progresses these punctiform or very small maculo-papular lesions increase in size and soon reach the diameter of a drop of water, when the disease is then called, for the purpose of convenience, "*psoriasis guttata*," from "gutta," a drop. When the lesions are of still larger size, resembling round, coin-sized patches, it is called *psoriasis nummularis*, and when many of these lesions all run together in one mass, it is termed *psoriasis diffusa*, from its diffuse character. Other names are also made use of to represent the different manifestations of the disease, but they are all one and the same disease, the names being applied for the sake of convenience to describe or designate the particular character met with in each case.

Ordinarily, the size and form of the lesions that you meet with in the average case that is presented to you will vary from the size of a silver ten-cent piece to a silver quarter of a dollar. Those are the sizes of the lesions generally met with, because the disease has generally passed from the first stages and conditions and becomes fully established before the case comes under your notice. Referring to the lesions as coin-shaped would naturally imply they are round or roundish. We have very many more important characteristics that refer exclusively to the lesion besides those I have mentioned. These coin-shaped lesions or patches are always of a reddish color, and if the part has not been interfered with, they are always coated over with thick scales—not crusts, remember—these scales are always of a white, shiny or silvery color. This is a very important characteristic to remember, because there is no other skin disease that has scales with this particular color or characteristic. When we remove them, which can be very readily done, as they are not very adherent, we find the red patch I described. We then observe little pin-point droplets of blood oozing out shortly after the scales are removed. This characteristic alone is almost pathognomonic of psoriasis, as it is not met with in any other disease of the skin. As regards the distribution of the disease, it always tends to symmetrical distribution—that is, the disease tends to involve both sides of the body equally. The lesions, of course, may be very scanty; there may not be over a half dozen or dozen lesions over the entire body, in which case we have some on both lower extremities, some on each upper extremity and so on, indicating that the disease tends to symmetrical distribution. Now, it not only tends to symmetrical distribution, but it also tends to be generally distributed; the one term is scarcely synonymous with the other. To say that the disease is symmetrically distributed might mean that both sides are alike involved, but it would not imply that it was a "generalized eruption," or involved the entire body, although the word "generalized" would imply symmetrical distribution. It tends to be a general eruption, and also symmetrically distributed.

Now as regards the skin between the lesions. You will recollect in certain other diseases, though we may have some circumscribed lesions, where the skin between them is not entirely healthy or free from redness. In psoriasis the lesions are accurately circumscribed, and the skin between and around them is always perfectly healthy. It is usually a very chronic disease—that is, it is liable to exist a very long time without treatment; and another feature is its liability to return after having once been cured by treatment. It is always dry from the first to last, never giving us any evidence of moisture in any form. The importance of this characteristic will be manifest when you contrast it with such a disease as eczema. We have almost no subjective symptoms—that is, we have no pain, itching or anything of that character—no symptoms experienced whatever by the feelings of the patient.

Now, while I stated the disease tends to universal or general distribution, there are certain localities that it favors, namely, the extensor surfaces. We almost invariably find more lesions on the extensor than on the flexor surfaces. We are almost sure to find some over the olecranon process, the patella, and sometimes on the sacrum. It almost never affects the palms and soles.

You see, then, it has a great many individual characteristics to identify it by, which you do not find in other skin affections. Another locality where we meet with it very seldom is the face. If we find it on the scalp, we very rarely find an eruption upon the face. Sometimes it will fringe the brow and extend down over the forehead a little, as in this plate I now show you. We very rarely encounter any constitutional derangement in the disease. It may occur in the most robust as well as the delicate, and perhaps oftener, if anything, in robust individuals than in delicate ones. So much, then, for the general characteristics of the disease.

Now, that you have got some knowledge of the symptoms, you may know what to look for and what to study in these cases. The little girl will be kind enough to pass around amongst you.

*Case.*—Miss Ada S—, æt. 7; eruption on body for six months; otherwise quite healthy. Sister has same disease; æt. 10. You notice first the round character of the lesions as she passes around; second, the white, shiny scales on each patch. In order to see the white, shiny character of the scales plainly, it will be necessary for you to scratch some of the lesions gently with your finger-nail; then it will be clearly manifest to you. You also notice the general distribution of the disease, affecting as it does both arms, both legs and sides of the neck. Having already had the disease some six months would, of course, indicate its chronic character. You see no evidence whatever of any moisture to which I have referred. It is entirely dry, and if you scratch off some of the scales you will find the characteristic pin-point droplets of blood. Notice, she complaims of no pain, itching or other subjective sensation; that is evident from the fact that you see no indications of scratching of the lesions by the girl herself. I think you can also notice in this case a preference for the extensor surfaces. By examining the case further you will notice the disease is well marked on the olecranon processes. While it is generally distributed over the arms and wrists, you find no patches whatever upon the palms and soles. There are only a few lesions on her neck. The children are both healthy, showing no indication whatever of constitutional disturbance.

ÆTIOLOGY—With reference to the ætiology of the disease, I need detain you but a few minutes. The fact is, very little is known of the cause or origin of the affection. As I stated, it may occur at any age, although it usually occurs in younger individuals. It may occur in both sexes; it will occur, regardless of hereditary predisposition. We now come, perhaps, to the most important point of the subject—the consideration of the diagnosis.

DIAGNOSIS.—As the disease is one characterized by many symptoms not met with in other diseases, the diagnosis is usually readily made by one familiar with it. In obscure cases, however, a careful study of the following diagnostic tables will enable one to make a diagnosis with positiveness:

DIFFERENTIAL DIAGNOSIS.

| PSORIASIS. | ECZEMA. |
|---|---|
| 1. No itching. | 1. Itching. |
| 2. No moisture. | 2. Moisture. |
| 3. Prefers extensor surfaces. | 3. Prefers flexor surfaces. |
| 4. General. | 4. Never universal. |
| 5. Symmetrical. | 5. Not. |
| 6. Shiny scales. | 6. Not. |
| 7. No crusts. | 7. Generally have. |
| 8. Lesions uniform. | 8. Multiform. |
| 9. Lesions round and well defined. | 9. Patches irregular and ill defined. |
| 10. Healthy skin between the lesions. | 10. Not. |
| 11. Pin-point bleeding on removing scales. | 11. Never so. |
| 12. Never palms and soles. | 12. Commonly so. |

| PSORIASIS. | SYPHILIS. |
|---|---|
| 1. No syphilitic history. | 1. Syphilitic history generally. |
| 2. Extensor surfaces. | 2. Not so. |
| 3. Lesions entirely covered by the scales. | 3. Not entirely covered by the scales; redness extends beyond the scanty covering. |

| PSORIASIS. | ECZEMA. |
| --- | --- |
| 4. Scales shiny and silvery. | 4. Not so much so. |
| 5. Never crusts. | 5. May have. |
| 6. Scales loose. | 6. More adherent. |
| 7. Redness bright or inflammatory in color. | 7. Dark or copper-colored. |
| 8. Pin-point bleeding on removal of scales. | 8. Not so. |
| 9. Les'ons uniform. | 9. Multiform. |
| 10. Never destructive. | 10. Generally is sooner or later. |
| 11. Never palms and soles. | 11. Frequently does. |
| 12. Never any moisture. | 12. Generally pus or othe moisture sooner or later. |

We frequently have to differentiate it from certain other diseases more or less peculiar to the scalp, as favus, tinea tonsurans and seborrhœa sicca.

In favus we never have scales, but crusts. The crusts are always, instead of being white and shiny as in psoriasis, of a bright or sulphur yellow color, as in the plate which I now show you. These crusts, characteristic of favus, are always cup-shaped. The hairs are brittle and broken off in favus, while their condition is normal in psoriasis. We find with the microscope the characteristic parasite of favus, which is absent in psoriasis. Favus is never a symmetrical disease—that is, we may find one or two patches or lesions on one side of the scalp and none on the other.

As differentiated from tinea tonsurans or ring-worm of the scalp, in psoriasis the disease is symmetrical, while in tinea tonsurans the disease is unsymmetrical. There may be a patch on one side of the head and none on the other. We never find the white, shiny scales in tinea tonsurans characteristic of psoriasis. We never find the coin-sized patches in tinea tonsurans that we do in psoriasis. We always find the hairs brittle and broken off, and some of them loose, in tinea tonsurans, but we never find this condition in psoriasis. We have also the discovery of the parasite in tinea tonsurans, which is absent in psoriasis.

From seborrhœa sicca of the scalp it may be differentiated from the entire absence of circumscribed lesions in that disease by the greasy character of the scales, and by its being more of a diffuse disease, sparing no particular portion of the scalp; the entire scalp is involved in the scaly process. No red patches with pin-point bleeding, etc.

*Treatment.*—As in most other scaly diseases, one of the first things to be done in adapting your local treatment is to get rid of the scales. This is best accomplished by first applying, say twice a day, some oleaginous preparation for the purpose of softening and loosening the scales, and taking a bath once a day, with plenty of soap, and a good brush or rough towel to remove the scales. This process should be repeated every day—the oleaginous preparation and the bath at night—for several days. Having gotten rid of the scale, the surface is prepared for treatment. Various remedies are used as local applications in psoriasis, among which I may mention chrysarobin, pyrogallic acid, naphthol and various mercurial preparations, as the citrine ointment, for instance. The chrysarobin is best applied in the form of an ointment—10 or 15 grains to the ounce of lard, vaseline, lanoline or rose ointmen t. Ten or fifteen grains, mixed with an ounce of ordinary rose ointment, make about as nice an application as a person could desire. Perhaps still better would be lanoline, it having a little more tendency to permeate the scales and tissues, and the object of the oleaginous preparation in this case is penetration rather than protection. If it was protection that was required, vaseline would be much better, inasmuch as it is a substance of a gummy, resinous character and seals the part over better. The chrysarobin ointment should be applied once or twice a day, and each patch requires its individual treatment—that is, the ointment should be applied to every patch on the body; then, in the course of several days or a week, this anointing and bath-soaping process must be again resorted to, because the scales will re-accumulate and the chrysarobin will not take effect properly until they are again removed by the process I have described. Instead of chrysarobin, pyrogallic acid may be substituted in the proportion of a half drachm to a drachm to the ounce. Ordinary citrine ointment may be used, which will also cure the disease. It may be used at full strength or diluted one-half or two-thirds. Another way of applying the chrysarobin is to have it suspended in collodion or a solution of gutta-percha, when it is then applied to each lesion. This solution forms a film, is well retained and prevents it from soiling the clothing.

Now, it is perhaps unnecessary to say much about the constitutional treatment any more than what I have already said, that if the constitution requires any regulation, to look after it by administering such remedies as will meet the indications.

The prognosis is good. The disease can almost invariably be cured in several weeks, but it is an affection that is very apt to return. You may cure it up and make the skin sound, but perhaps the next season you will find the disease has returned, more especially in cold weather.

